# Identifying Angiogenic Factors in Pediatric Choroid Plexus Papillomas

**DOI:** 10.3390/neurosci6030076

**Published:** 2025-08-11

**Authors:** Nurfarhanah Bte Syed Sulaiman, Sofiah M. Y. Sng, Khurshid Z. Merchant, Lee Ping Ng, David C. Y. Low, Wan Tew Seow, Sharon Y. Y. Low

**Affiliations:** 1 Neurosurgical Service, KK Women’s and Children’s Hospital, Singapore 229899, Singapore; 2 Department of Pathology and Laboratory Medicine, KK Women’s and Children’s Hospital, Singapore 229899, Singapore; 3 Department of Neurosurgery, National Neuroscience Institute, Singapore 308433, Singapore; 4 SingHealth Duke-NUS Neuroscience Academic Clinical Program, 11 Jalan Tan Tock Seng, Singapore 308433, Singapore; 5 SingHealth Duke-NUS Pediatrics Academic Clinical Program, 100 Bukit Timah Road, Singapore 229899, Singapore

**Keywords:** angiogenesis, choroid plexus papilloma, cytokines

## Abstract

(1) Background: Choroid plexus papillomas (CPPs) are rare brain tumors that tend to occur in very young children. Mechanisms of CPP development remain unelucidated. Separately, the process of angiogenesis has been implicated in other primary brain tumors. We hypothesize that angiogenesis is a hallmark of CPP biology. This study aims to identify and validate angiogenic factors in CPPs. (2) Methods: Cerebrospinal fluid (CSF) and CPP tumor samples are collected. A multiplex immunoassay panel is used to identify differentially expressed cytokines in the CSF samples. Concurrently, patient-derived primary cell cultures and their supernatants are derived from CPP samples. Targeted proteome blot arrays and human umbilical vein endothelial cell (HUVEC) angiogenesis assays are used for validation studies. (3) Results: CSF profiling showed higher expressions of VEGF-A, MCP-1, MMP-1, TNF-α, and CD40L in CPP patient samples versus non-tumor controls. Next, assessment via online protein–protein network platforms reports that these cytokines are associated with endothelial cell regulation. Using an angiogenesis-focused approach, CPP-derived cell lines and supernatants showed similarly higher expressions of VEGF, MCP-1, and MMP-1. Next, sprouting of nodes and tubule formation were observed in HUVEC angiogenesis assay cultures when conditioned CPP cell culture media was added. (4) Conclusions: This proof-of-concept study demonstrates potential to explore angiogenesis in CPP.

## 1. Introduction

Choroid plexus papillomas (CPPs) are rare, primary brain tumors of neuroectodermal origin with a predilection to occur within the first 2 years of life [1,2]. They account for less than 1% of all intracranial brain tumors [2,3]. Patients often present with life-threatening symptoms of hydrocephalus from a combination of cerebrospinal fluid (CSF) overproduction from neoplastic tufts of villi within the ventricular system that produces cerebrospinal fluid (CSF), physical obstruction, and a local mass effect from the tumors themselves [3]. Gross total resection is the recommended mainstay of treatment. Nonetheless, surgery can be technically difficult due to their deep intraventricular locations and innately high vascularity [4,5,6]. Historically, intraoperative blood loss is a significant source of morbidity and mortality in the surgical treatment of hyper-vascular CPP in young children with small blood volumes [7,8]. Concurrently, intra-tumoral bleeding has been reported to occur within CPP—adding on another layer of complexity to the surgery [3]. Of clinical significance, a subset of CPPs have a propensity for tumor recurrence, malignant progression, and/or metastasis [9]. Currently, the definitive role of adjuvant therapies and optimal management of these tumors are still matters of debate [10]. Owing to the infrequency of CPP, in-depth mechanisms of CPP behaviors remain unelucidated. Early studies suggest immune-mediated factors to be a cause for their pathogenesis [11,12]. However, this has not been definitively proven.

In recent years, the role of cytokines has been increasingly demonstrated in different cancers [13,14,15]. Broadly speaking, cytokines consist of a heterogeneous group of small, secreted proteins released by cells under inflammatory conditions and contribute to the pathophysiology of various diseases, including cancer [16]. Individually, each has a specific effect on the interactions and communications between cells [17]. Cytokines and their immune-related partners are known to facilitate various cell–cell communications within the tumor microenvironment, acting on autocrine and/or paracrine pathways on both malignant and non-malignant cells [15,18]. Identification of these cytokines is believed to be fundamental for selecting therapeutic targets in affected patients [15,19]. We are now aware that the cytokine-rich CSF is a good medium to study biological changes in different brain tumors [19,20]. In particular, cytokines in CSF provide information about the protein–protein interaction between neoplastic cells and their microenvironment to provide regulatory support for oncogenic processes such as tumor growth, angiogenesis, invasion, and metastasis [13,20]. Under such circumstances, CPP’s innate ability to produce CSF presents a unique opportunity to examine the latter in detail. By assessing biomolecular networks constructed via computational biology, we hope to identify in silico protein–protein association networks related to CPP biology [21,22]. Recognizing these protein–protein associations is important to delineate their functional associations—meaning that two proteins that are deemed to be functionally associated may jointly contribute toward a specific cellular process [15,21,23].

As the normal choroid plexus tissue is known to harbor inherently high expression of vascular endothelial growth factor (VEGF) [24,25] and due to intraoperative observations of CPPs’ vascularity, we postulate that CPPs are enriched with angiogenic factors that promote aberrant growth of new blood vessels in their tumor microenvironment. We designed an exploratory, pre-clinical study to investigate this hypothesis via a proteomic-based approach [26]. Put together, this proof-of-concept study aims to, first, identify secretory factors in CPPs and, second, elucidate their potential roles via in vitro functional assays.

## 2. Materials and Methods

### 2.1. Study Design and Patient Selection

This was an ethics-approved, retrospective study based at the KK Women’s and Children’s Hospital (SingHealth CIRB Reference 2014/2079). Patients with a histologically proven diagnosis of CPP were included. Exclusion criteria encompassed the following: patients with other brain tumor types, insufficient CSF/tumor specimens, and/or incomplete medical records.

### 2.2. Multiplex Immunoassay for CSF Samples

The Invitrogen Immune Monitoring 65-Plex Human ProcartaPlex™ Panel (Thermo Fisher Scientific, Waltham, MA, USA) was used to measure cytokine concentration in the CSF collected from patients. All standards and samples were prepared according to the manufacturer’s instructions with data acquired using the Luminex 200™ System. The cytokine panel consisted of the following analytes: APRIL, BAFF, BLC, NGF-b, CD30, ENA-78Eotaxin, Eotaxin-2, Eotaxin-3, FGF-2, Fractalkine, G-CSF, GM-CSF, GRO-a, HGF, IFN-a, IFN-g, IL-1a, IL-1b, IL-10, IL-12p70, IL-13, IL-15, IL-16, IL-17A, IL-18, IL-2, IL-2R, IL-20, IL-21, IL-22, IL-23, IL-27, IL-3, IL-31, IL-4, IL-5, IL-6, IL-7, IL-8, IL-9, IP-10, I-TAC, LIF, MCP-1, MCP-2, MCP-3, MCSF, MDC, MIF, MIG, MIP-1a, MIP-1b, MIP-3a, MMP-1, CD40L, SCF, SDF-1a, TNF-a, TNF-b, TNF-RII, TRAIL, TSLP, TWEAK, and VEGF-A. Briefly, samples were diluted in a 1:4 ratio with universal assay buffer with a final volume of 50 μL loaded into each well in duplicates. A 5-point-log curve was generated for standards of known concentration and data analysis was performed using the Procartaplex Analysis application. Technical duplicates were analyzed for each condition (25 to 50 μL/sample) and read through the Bio-Plex Multiplex System (Bio-Rad, Hercules, CA, USA) following standard procedures. Data analysis was performed with MAGPIX xPONENT 4.2 software (Luminex Corporation, Austin, TX, USA).

### 2.3. Patient-Derived Tumor Cell Cultures and In Vitro Experiments

Our primary cell culture technique has been previously described [14]. Briefly, histologically confirmed CPP tissues were finely minced, washed in Dulbecco’s Phosphate-Buffered Saline (Naclai Tesque, Kyoto, Japan), before being plated onto 60 mm tissue culture dishes (Corning^®^, New York, NY, USA). The cell culture media contained DMEM-F12A with L-Glutamine (Capricorn Scientific, Ebsdorfergrund, Germany) supplemented with 10% fetal bovine serum (Capricorn Scientific, Ebsdorfergrund, Germany) and 1% actinomycin (Naclai Tesque, Kyoto, Japan). All cultures were maintained in a standard 37 °C, humidified incubator with 95% air and 5% CO_2_. Viable cells were trypsinized, expanded, and seeded at 1 × 10^6^ cells per 10 cm dish in 10 mL of culture media. Verification of molecular markers reminiscent of original CPP tumors were examined via real-time quantitative polymerase chain reaction (qPCR) [3,7,27,28,29] (Appendix A). Only cultured cells at fewer than 8 passages were used for experiments in this study. Conditioned cell culture media (in the form of cell culture supernatant) was collected from cell cultures upon attaining a growth confluency of 70%. Collected cell culture supernatants were stored in small working aliquots at −80 °C until further use. Frozen working aliquots were thawed overnight at 4 °C and subjected to centrifuging at 13,200 rpm for 5 min prior to use for subsequent experiments.

### 2.4. Proteome Blot Array

The proteome blot array used was the Human Angiogenesis Antibody Array (Abcam, Cambridge, UK) that targets 43 proteins. The following technique for this array has also been validated in our previous study [13]. In short, cell culture supernatants were collected and centrifuged at 14,000 rpm for 5 min, prepared, and incubated with a pre-configured antibody array in the form of an immunoblot, as per manufacturer’s instructions. Following that, chemiluminescent imaging was performed using the ChemiDoc™ Touch Imaging System version 1.2 (Bio-Rad, Hercules, CA, USA) and processed via ImageLab version 6.0.1 (Bio-Rad, Hercules, CA, USA). Membrane blot signal intensities were quantified by ImageJ software version 1.52a (NIH, Bethesda, MD, USA) after normalization to internal controls. Next, ratios of the respective cytokine expressions and internal control standards were analyzed. The blots shown in this study were performed in up to 5 min exposures.

### 2.5. Referencing Protein–Protein Biomolecular Networks In Silico

Online resources dedicated to organism-wide protein association networks were used to look for protein–protein interaction pathways based on our experimental results. These included the STRING (Search Tool for the Retrieval of Interacting Genes/Proteins; https://string-db.org/ (accessed on 28 April 2025)) and BioGRID (Biological General Repository for Interaction Datasets; http://thebiogrid.org/ (accessed on 28 April 2025)) databases [21,22]. Briefly, proteins of interest are input into its online platform. Here, they are tested for pathways that have a skewed distribution on either end of the user’s ranked input. Significant pathways that are simultaneously enriched on the user’s input are reported and can subsequently be examined interactively [21].

### 2.6. Assessment of In Vitro Angiogenesis: HUVEC Functional Assay

The human umbilical vein endothelial cell (HUVEC) tubule-formation angiogenic assay assesses the ability of HUVECs to form three-dimensional tubular structures when cultured on a gel of growth factor-reduced basement membrane extracts. Based on the premise that the conditioned cell medium from primary CPP cell lines contains secreted angiogenic factors and cytokines, the HUVEC experiment offers functional validation of our hypothesis. Briefly, Geltrex™ LDEV-Free Reduced Growth Factor Basement Membrane Matrix (ThermoFisher Scientific, Waltham, MA, USA) was added to pre-chilled 96-well plates and allowed to polymerize at 37 °C for 1 h. Here, fresh DMEM-F12A supplemented with 20 ng/mL of FGF-2 was used as the positive control while 40 mM Suramin was used as a negative control. The HUVECs were cultured to 70% confluency and collected. Next, they were suspended in 100 µL of conditioned medium (1:2 or 1:4) and seeded onto 96-well plates (2 × 10^4^ cells/well) containing Matrigel. Following an 18 h incubation, the capillary-like structures were observed under a light microscope, and pictures of 3 to 5 visual fields per well were taken. Various dimensions of the tubular structures were captured and quantified using ImageJ software version 1.2 (NIH, Bethesda, MD, USA) [30]. Subsequent re-assessment was performed via Wimasis (https://www.wimasis.com/en/ (accessed on 28 April 2025)) [31].

### 2.7. Statistical Analysis

Statistical analyses and graphs were generated using GraphPad Prism version 10.5.0 (GraphPad Software, La Jolla, San Diego, CA, USA). Data are presented as mean ± standard deviation (SD). Analyses were performed using either the Analysis of Variance (ANOVA) test or Student’s *t*-test. For the purposes of this study, differences between sample means were considered statistically significant when the *p*-value was less than 0.05 (*) or less than 0.001 (**).

## 3. Results

### 3.1. Summary of Study Workflow and Findings

**Figure 1:**
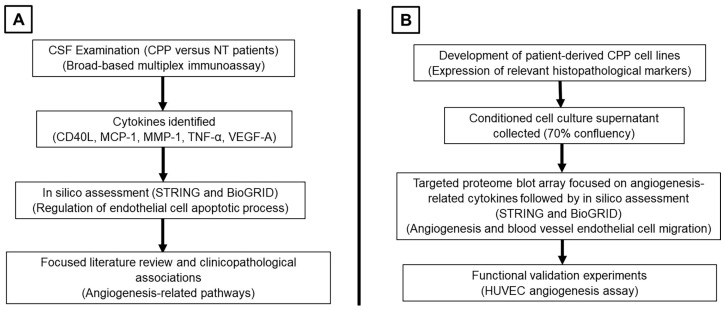
(**A**) Part 1 of the study for the examination of CSF from CPP to identify cytokines of interest. (**B**) Part 2 of the study using an in vitro approach to functionally validate findings from Part 1. (abbreviations: CSF = cerebrospinal fluid, CPP = choroid plexus papilloma, NT = non-tumor, HUVEC = human umbilical vein endothelial cell).

This study is divided into two parts. First, CSF from 5 histologically confirmed CPP patients were interrogated as part of the multiplex immunoassay and compared with a cohort of CSF from 3 non-tumor patients (2 congenital hydrocephalus and 1 post-traumatic hydrocephalus) (Appendix B). Here, selective cytokines common across samples with differentially expressed protein expressions were identified. These findings were then input into online databases to identify relevant protein–protein interactions from known networks. Following that, an in vitro approach using patient-derived CPP cell lines was designed to verify our results (Figure 1).

### 3.2. Selective CSF Cytokines Are Differentially Expressed Between CPP Versus Non-Tumor Samples and They Demonstrate Biomolecular Associations In Silico

**Figure 2:**
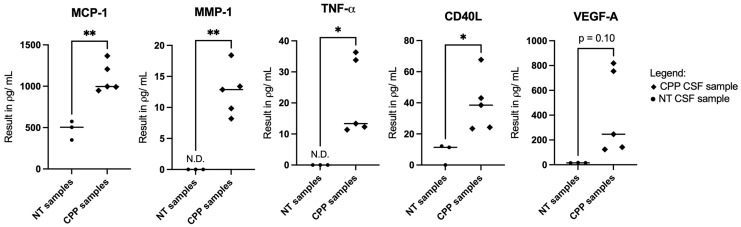
Graph results of CSF profiling for selected cytokines in CPP patients versus non-tumor controls. These include MCP-1, MMP-1, TNF-α, CD40L, and VEGF-A. Statistically significant analyses are represented when the p-value was less than 0.05 (*) or less than 0.001 (**). (Abbreviations: CPP = choroid plexus papilloma, N.D. = not detectable, NT = non-tumor, TNF-α = Tumour Necrosis Factor Alpha).

**Figure 3:**
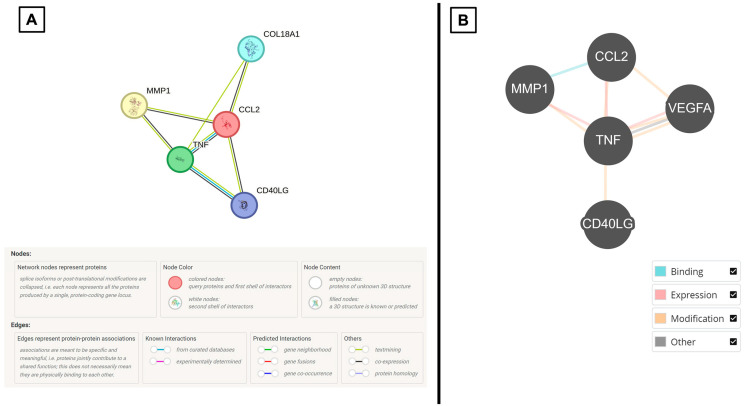
Summary diagrams of results of input CSF cytokines and their associations with corresponding legends generated from (**A**) STRING (unclustered) and (**B**) BioGRID.

Based on the multiplex immunoassay profiling, we observed that there were higher expressions of VEGF-A, MCP-1, MMP-1, TNF-α, and CD40L in the CSF samples of CPP patients in comparison to non-tumor patients (Figure 2 and Table 1). Following that, STRING and BioGRID online platforms show that these cytokines of interest are strongly associated with the regulation of endothelial cells (Figure 3 and Table 2). From a clinical perspective, this is relevant because the endothelium functions to control blood fluidity, platelet aggregation, and vascular tone, and it has major roles in the regulation of inflammation and angiogenesis [21].

### 3.3. Patient-Derived CPP Cell Lines Secrete Cytokines That Are Associated with Vascular-Related Pathways

**Figure 4:**
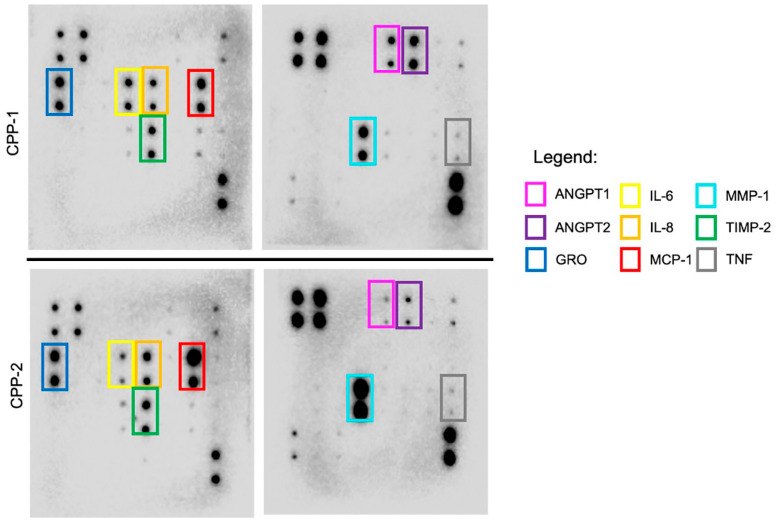
Proteome array blots of the conditioned cell culture supernatant in 2 patient-derived CPP cell lines (CPP-1 and CPP-2). Cytokines of interest are highlighted in colored outlines and labeled in the corresponding legend. (Original proteome blots and layout of array are available in Supplementary File S1).

**Figure 5:**
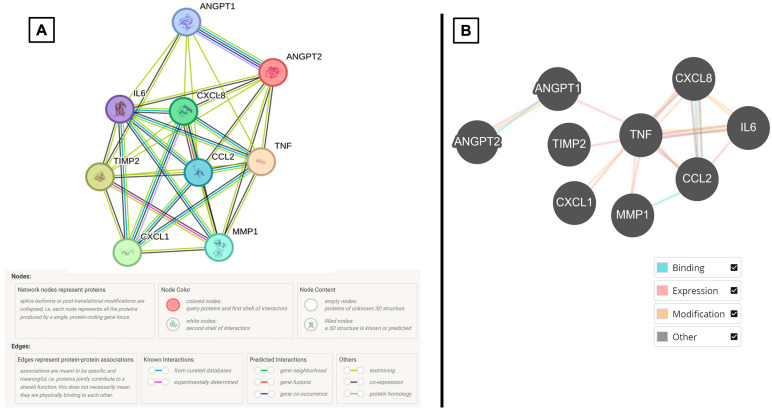
Summary diagrams of results of input CSF cytokines and their associations with corresponding legends generated from (**A**) STRING (unclustered) and (**B**) BioGRID.

Two patient-derived CPP cell lines were investigated for this part of the study. Here, a targeted proteome array focused on angiogenesis-related cytokines was used. We observed that conditioned cell culture supernatant harvested from both cell lines have similar secretory cytokine profiles. They demonstrate higher expressions of MCP-1, MMP-1, and TNF, similar to the CSF of CPP patients. Of note, there were other cytokines in the cell culture supernatant that showed higher expression. These included CXCL1, IL-6, IL-8, TIMP-2, ANGPT1, and ANGPT2 (Figure 4 and Table 3). As per our previous CSF cytokine workflow, these findings were input into the STRING and BioGRID platforms whereby they show involvement in pathways associated with angiogenesis and endothelial cell biology (Figure 5 and Table 4).

### 3.4. Secreted Cytokines from CPP Cell Lines Demonstrate In Vitro Angiogenesis by HUVEC Functional Assay

**Figure 6:**
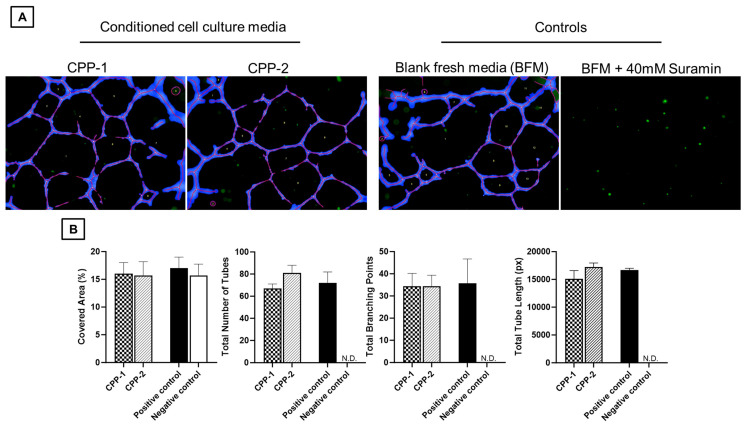
Cultured HUVECs were seeded on Matrigel-lined plates and incubated with conditioned media from CPP-1 and CPP-2 cells. At the timepoint of 18 h, photographs of newly formed tubular and branching structures were taken under a light microscope. (**A**) Representative images of HUVECs forming tubular and branching structures (outlined in purple) [images generated by Wimasis, (https://www.wimasis.com/en/ (accessed on 28 April 2025)) [31]]. (**B**) Graphical quantification of the HUVEC assay findings in sequence for covered area (%), total number of tubes, total branching points, and total tube length (pixels). (Abbreviations: N.D. = not detected).

To validate the above results, the HUVEC assay was used. Here, we observed the development of angiogenic sprouting (‘nodes’) and capillary lumen formation (‘tubes’) formed by confluent HUVEC cultures where conditioned CPP cell culture media was added (Figure 6).

## 4. Discussion

### 4.1. Overview of Choroid Plexus Papillomas in Children

To date, the pathogenesis of CPP remains poorly understood. These primary brain tumors are postulated to arise from differentiated epithelial tissue of neuroectodermal origin [7,32]. Current treatment of choroid plexus tumors, including CPP, is based on limited evidence [32]. There is no definitive clinical trial to suggest that adjuvant therapies significantly improve overall survival for CPP patients [32]. Reasons for the comparatively less research on CPP are, first, that these are very rare tumors and, second, that complete surgical resection is usually curative [33]. Although CPPs are presumed to be low-grade and so-called ‘benign’ entities, they have been occasionally shown to progress to high-grade lesions, metastasize into the CSF, and cause neoplastic seeding into distal sites [4,9,34,35,36]. Sporadic cases of CPP undergoing malignant transformation have been reported in the literature [5,35,36,37,38]. In addition, tumor recurrence has also been reported in up to 50% of patients after surgical intervention [7,39,40]. Overall, we are now aware that the family of choroid plexus tumors are uniquely complex because histological appearances may not necessarily predict their behavior, in spite of an established grading scheme. To complicate matters, CPPs are highly vascularized. For the operating neurosurgeon, intraoperative massive bleeding and serious consequences of dangerous hemodynamic disturbances in pediatric CPP patients are established risks [8]. Preoperative embolization has been proposed as an adjunct to surgical resection; however, it is rarely successful due to the small caliber of feeding vessels, especially in the very young [4,41,42]. Furthermore, the current evidence for impactful adjuvant therapies in CPP patients remains unestablished [32].

In recent years, molecular studies have identified differential expressions of specific transcription factors in CPP tumors, in comparison to normal choroid plexus. Notable examples include Twist-related protein 1 (TWIST1), Wnt inhibitory factor 1 (WIF1), B-cell lymphoma 2-associated transcription factor 1 (BCLAF1), and so forth [7,43]. At the same time, a combination of commonly occurring chromosomal duplications and deletions has also been recognized [7]. Put together, there is emerging evidence that CPP has distinct molecular aberrations that may be targetable for treatment in the future. Nonetheless, in-depth mechanistic studies on CPP biology, particularly those focused on secretory factors, are sparse in the literature at the time this writing.

### 4.2. The Relevance of Angiogenic CSF Cytokines in CPP Tumors

The choroid plexus is an integrated epithelial–endothelial convolute composed of the epithelium, stroma, and a rich vascular supply. This important structure is responsible for CSF production and forms the blood–CSF barrier [44]. Under physiological conditions, angiogenesis facilitates the formation of new blood vessels from pre-existing vasculature that are essential for cell development and tissue growth [45]. This process is regulated by a plethora of various other proteins [45]. An example is VEGF-A which plays an important role in maintaining CSF homeostasis and the function of the normal choroid plexus [46]. Paradoxically, upregulated angiogenesis is a hallmark of cancer development [47]. Briefly, angiogenic factors bind to receptors at the surface of endothelial cells under hypoxic conditions which leads to their dilatation and activation. At the same time, hypoxia upregulates the expression of some proteases that induce basement membrane degradation and pericyte detachment. Next, the stimulated motile endothelial cells migrate, proliferate, and form new blood vessels [47,48]. Following that, the basement membrane is reformed and pericytes are recruited. Finally, the different blood vessels merge, concluding the formation of the individual tumor’s vasculature [48]. The most well-studied angiogenesis promoter is VEGF whereby the monoclonal antibody targeting it, bevacizumab, is an approved anti-angiogenic drug [49]. To date, its use in CPP has only been previously reported in a single adult case as salvage treatment [6]. However, temporal cancer research has highlighted that targeting VEGF alone is likely insufficient in the treatment of primary brain tumors [49,50]. Alternative angiogenesis pathways, vasculogenic mimicry mechanisms, upregulation of other pro-angiogenic factors, and so forth have been cited to take effect when VEGF is targeted in neoplasms [51,52,53,54,55]. We are now aware that they tend to provide short-term relief from tumor growth before resistance occurs, resulting in modest survival benefits [56]. Under such circumstances, the design of novel therapeutics should endeavor to incorporate targeting multiple targets in the angiogenesis pathways and beyond.

In our study, we note that there is consistently high expression of MCP-1 and MMP-1. Broadly speaking, the former is a ubiquitous chemotactic factor which typically recruits macrophages during an immune response to regulate the activity of monocytes, memory T lymphocytes, and natural killer (NK) cells [57,58]. Working with its cell surface receptors, MCP-1 impacts a myriad of conditions [13,57]. In the context of angiogenesis, several studies have demonstrated that MCP-1 has direct and indirect effects in promoting this process in numerous diseases, including cancer [59,60,61,62,63]. Broadly speaking, MCP-1 is expressed in a variety of cell types including vascular endothelial cells following induction by various stimuli, including TNF-α [64]. Of note, TNF-α has been shown to upregulate MCP-1 expression and secretion during the inflammatory process of endothelial dysfunction in pre-clinical studies of type 2 diabetes [65]. However, the exact mechanism by which TNF-α stimulates expression of the MCP-1 gene is not fully established [64]. On the other hand, MMP-1 is an integral member of a zinc-dependent endopeptidase family that regulates the degradation of the extracellular matrix (ECM) and basal membrane [66]. Clinical studies have also identified MMPs as prognostic markers and therapeutic targets in multiple neoplasms [67,68]. Essentially, MMP-1 functions as an interstitial collagenase that is active in extracellular matrix and vascular remodeling, angiogenesis, and tumor progression [69,70]. Some studies show that VEGF can induce MMP-1 expression in human endothelial cells under physiological conditions by modulating ECM remodeling and microvascular permeability [71,72,73]. Additional reported associations between MMP and VEGF variants include the promotion of malignant cell growth, chemoresistance, and angiogenesis in retinoblastoma tumors [74].

At this juncture, we reiterate that exact mechanisms on how VEGF-A, MCP-1, and MMP-1 combinatorially mediate angiogenic processes in CPP tumors are still unelucidated. Nonetheless, we believe that the insights from pertinent protein–protein interaction networks can provide direction for the next step of this project. In this modern era of medicine, bioinformatics have enabled modeling and simulation of sub-cellular and cellular processes via methods from dynamical systems theory [75]. Contemporary translational research often relies on such applications to streamline the analysis of datasets generated from in-house studies. These in silico models are facilitated by rapidly advancing experimental and analytical tools that generate information-rich, high-throughput biological data [75]. More importantly, the models encode and test hypotheses about cell functions and disease pathophysiology of disease, therefore contributing to identification of new drug targets and drug design [75]. Therefore, we believe that applying in silico information to representative cellular models is a step forward in better understanding of knowledge gaps in CPP biology. In the context of our study, these findings help justify further characterization of the identified cytokines for affected patients.

### 4.3. Study Critique and Future Work

The authors acknowledge that there are noteworthy limitations that should be highlighted. First and foremost, our sample size is small. This is not unexpected, as CPPs are rare tumors that are more prevalent in children. In addition, the availability of age-matched experimental controls is not straightforward, as most commercial CSF biobanks tend to have adult samples or pooled patient samples. Although our results may potentially have translational value, they remain preliminary in this stage. As this is an exploratory study, the use of multiplex platforms offers advantages of high throughput and good sensitivity for lower sample volumes compared to traditional enzyme-linked immunosorbent assays (ELISAs). In reality, higher volumes of CSF required per patient are likely challenging to extract from very young children in a clinical setting. Moreover, our chosen platform has been validated by previous studies for its ability to simultaneously measure multiple secreted cytokines from a single sample for comprehensive analysis [13,76,77].

At this juncture, we believe our findings provide precedence to understand CPP in-depth, especially in regard to, first, anti-angiogenic targets to aid resection safety in very young children and, second, the subset of tumors that are recurrent and or metastatic. As part of a wider prospective study in pediatric brain tumors at our institution, efforts are ongoing to recruit more CPP patients and their biomaterial for research. In addition, we advocate in-depth studies in an extended population, and global collaborative efforts for better understanding of this rare primary brain tumor.

## 5. Conclusions

We therein report this proof-of-concept study focused on understanding pediatric CPPs via an in vitro approach. Our findings strengthen the potential to investigate the role of angiogenesis and its associated factors in CPP from a translational perspective.

## Figures and Tables

**Table 1:** Cytokines found to be differentially expressed in the CSF of CPP patients versus non-tumor patients (note: cytokines common to Table 1 and Table 3 are highlighted in italics).

Cytokine Abbreviation (Other Aliases)	Protein Name
*MCP-1* (*CCL2*)	*Monocyte chemoattractant protein 1*
MMP1 (Interstitial collagenase, fibroblast collagenase)	*Matrix metalloproteinase-1*
*TNF-α* (*TNF*, *cachexin*, *cachectin*)	*Tumor necrosis factor*
VEGF-A (VEGF)	Vascular endothelial growth factor A
CD40L (CD40LG, CD154)	CD40 ligand

**Table 2:** Top relevant biological processes and enriched pathways reported based on input cytokines from 2 independent online resources for CSF results.

Online Resource	Biological Process/Enriched Pathways
STRING	Regulation of endothelial cell apoptosis process Cellular extravasation
BioGRID	Endothelial cell apoptotic process regulation

**Table 3:** Cytokines differentially secreted in conditioned cell culture supernatant versus blank fresh media (note: cytokines common to Table 1 and Table 3 are highlighted in italics).

Cytokine Abbreviation (Other Aliases)	Protein Name
*MCP-1* (*CCL2*)	*Monocyte chemoattractant protein 1*
MMP1 (interstitial collagenase, fibroblast collagenase)	*Matrix metalloproteinase-1*
*TNF-*α (*TNF*, *cachexin*, *cachectin*)	*Tumor necrosis factor*
TIMP-2	Tissue inhibitor of metalloproteinases 2
CXCL1 (C-X-C motif chemokine ligand 1, GRO-α, GRO-1)	Growth-regulated oncogene alpha
IL-6	Interleukin 6
IL-8 (CXCL8, MDNCF)	Interleukin 8
ANGPT1 (ANG1)	Angiopoietin 1
ANGPT2 (ANG2)	Angiopoietin 2

**Table 4:** Top relevant biological processes and enriched pathways reported based on input cytokines from 2 independent online resources for conditioned cell culture supernatant results.

Online Resource	Biological Process/Enriched Pathways
STRING	Tie signaling pathway Angiogenesis Maintenance of blood–brain barrier
BioGRID	Blood vessel endothelial cell migration Vasculature development Endothelial cell apoptotic process regulation

**Figure A1:**
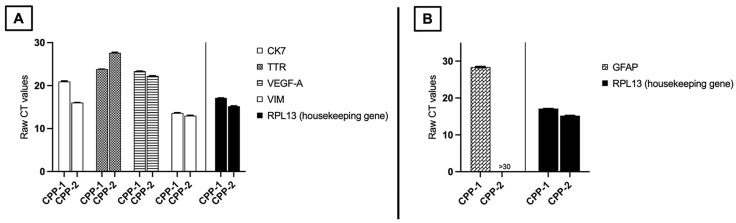
(**A**,**B**) Graphs depicting raw CT values for genes of interest in CPP tumors as curated from standard clinical diagnosis and the literature [3,7,27,28,29]. Not unexpectedly, both samples demonstrated equivocal GFAP expression. This is a known phenomenon postulated to be secondary to the variable ependymal differentiation in some CPPs and not others [29] (abbreviations: CK7 = cytokeratin 7, CT = threshold cycle, GFAP = glial fibrillary acidic protein, TTR = transthyretin, VEGF-A = vascular endothelial growth factor A, VIM = vimentin, RPL13 = ribosomal protein L13).

## Data Availability

The original contributions presented in this study are included in the article. Further inquiries can be directed at the corresponding author.

## Supplementary Materials

The following supporting information can be downloaded at: https://www.mdpi.com/article/10.3390/neurosci6030076/s1, Supplementary File S1 consists of Figure S1: Full length proteome array blots (a,b) of conditioned cell culture supernatant from CPP-1 sample; Figure S2: Full length proteome array blots (a,b) of conditioned cell culture supernatant from CPP-2 sample.

## Appendix A

### Real-Time Quantitative Polymerase Chain Reaction (qPCR) for mRNA Expression

Total RNA was extracted using TRIzol reagent (Life Technologies, Waltham, CA, USA) and chloroform (Sigma-Aldrich, Burlington, MA, USA) and purified using the RNeasy Plus Mini Kit (Qiagen, Hilden, Germany) according to the manufacturers’ instructions. The GoScriptTM Reverse Transcription Mix, Oligo(dT) (Promega, Madison, WI, USA), was used to synthesize cDNA for qPCR amplification using 500 ng of RNA sample per reaction according to the manufacturer’s instructions. Quantitative real-time-PCR was performed on the CFX96TM Real-Time Detection System (Bio-Rad Laboratories, Herculais, CA, USA) using SsoAdvanced Universal SYBR Green Supermix (Bio-Rad Laboratories, Herculais, CA, USA) according to the manufacturer’s instructions, using 50 ng of cDNA and 2 μL of 10 μM primers per reaction. All reactions were carried out in triplicates. Threshold cycles (Ct) were calculated using the CFX Manager™ Software (Version 3.1) (Bio-Rad Laboratories, Herculais, CA, USA). For the purposes of this experiment, Ct values below 30 were accepted. Data are presented as mean ± standard deviation (SD). Graphs were generated using GraphPad Prism version 10.5.0 (GraphPad Software, La Jolla, San Diego, CA, USA).

**Table A1:** Summary of primers used in qPCR.

Gene of Interest	Forward Primer Sequence	Reverse Primer Sequence
Cytokeratin 7 (CK7)	GAAAGGCTGCTGATGACACC	TCAGTTGTGAGCCCATGCAG
Glial fibrillary acidic protein (GFAP)	CTGGAGGTTGAGAGGGACAA	CAGCCTCAGGTTGGTTTCAT
Transthyretin (TTR)	GAAAGGCTGCTGATGACACC	TCAGTTGTGAGCCCATGCAG
Vascular endothelial growth factor A (VEGF-A)	TTGCCTTGCTGCTCTACCTCCA	GATGGCAGTAGCTGCGCTGATA
Vimentin (VIM)	TGCAGGAGGCAGAAGAATGG	ATTTCACGCATCTGGCGTTC
Ribosomal protein L13 (RPL13) *	CATAGGAAGCTGGGAGCAAG	GCCCTCCAATCAGTCTTCTG

* Used as housekeeping gene.

## Appendix B

**Table A2:** A summary of clinicopathological details of patients whose CSF samples were interrogated via the Invitrogen Immune Monitoring 65-Plex Human ProcartaPlex™ Panel (ThermoFisher Scientific, Waltham, MA, USA) depicted in Section 2.2. Of note, primary cell lines were cultured from the excess tumor specimens of CPP-1* and CPP-2*. These were subsequently used in experiments described in Section 2.3, Section 2.4, Section 2.5 and Section 2.6 (abbreviations: CPP = choroid plexus papilloma; CSF = cerebrospinal fluid; GTR = gross total resection; N.A. = not applicable; STR = subtotal resection).

**S/N**	**Age (Months)**	**Tumor Location**	**Diagnosis**	**WHO CNS Grade**	**Treatment**
CPP-1*	10	Right atrium	CPP	1	GTR
CPP-2*	84	Fourth ventricle	Atypical CPP	2	STR
CPP-3	24	Left atrium	CPP	1	GTR
CPP-4	15	Right frontal horn	CPP	1	GTR
CPP-5	2	Right atrium	Atypical CPP	2	GTR
NT-001	2.5	Congenital hydrocephalus	N.A.	N.A.	CSF diversion
NT-002	3	Congenital hydrocephalus	N.A.	N.A.	CSF diversion
NT-003	72	Post-traumatic hydrocephalus	N.A.	N.A.	CSF diversion
