# Supplementary material for: Identifying Angiogenic Factors in Pediatric Choroid Plexus Papillomas
Source: NeuroSci. 2025 Aug 11;6(3):76. doi: 10.3390/neurosci6030076 (PMC12372068; doi:10.3390/neurosci6030076)
Supplement: Supplementary file 1 [file neurosci-06-00076-s001.zip › neurosci-3649093-supplementary.pdf]

Supplementary File S1

Figure S1: Full length proteome array blots (**a and b**) of conditioned cell culture supernatant from CPP-1 sample. Layout of the Human Angiogenesis Antibody Array (Abcam, Cambridge, UK) is available on: https://www.abcam.com/human-angiogenesis-antibody-array-membrane-43-targets-ab193655.html.

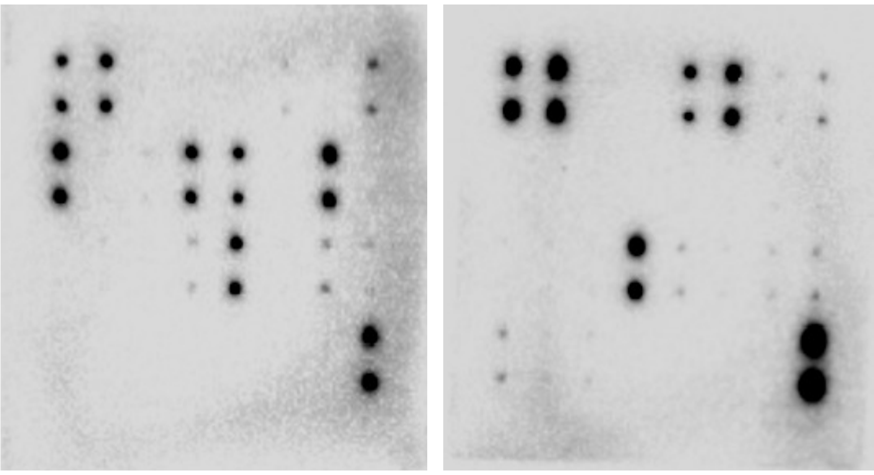

(a) **(b)**

Figure S2: Full length proteome array blots (**a and b**) of conditioned cell culture supernatant from CPP-2 sample. Layout of the Human Angiogenesis Antibody Array (Abcam, Cambridge, UK) is available on: https://www.abcam.com/human-angiogenesis-antibody-array-membrane-43-targets-ab193655.html.

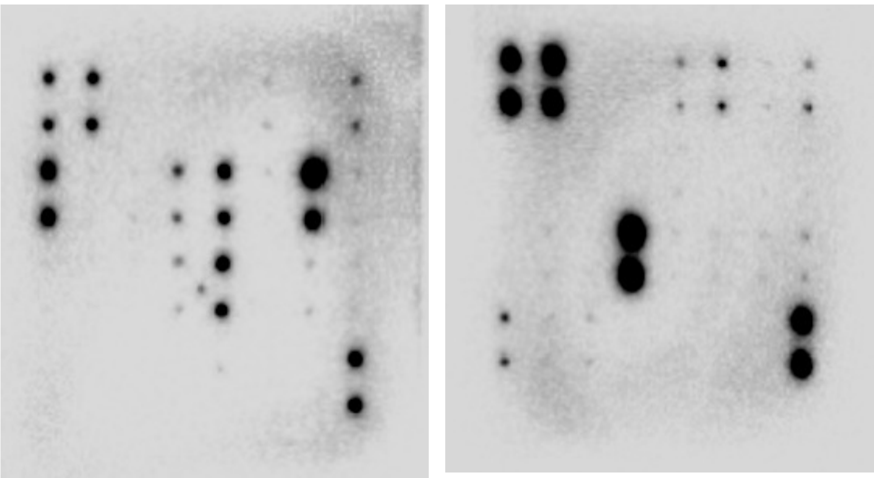

(a) **(b)**
